# Association of Peri‐Implant Mucosa Dimensions With Emergence Profile Angles of the Implant Prosthesis

**DOI:** 10.1002/cre2.939

**Published:** 2024-07-23

**Authors:** Piboon Rungtanakiat, Natchaya Thitaphanich, Martin Janda, Franz Josef Strauss, Mansuang Arksornnukit, Nikos Mattheos

**Affiliations:** ^1^ Department of Prosthodontics, Faculty of Dentistry Chulalongkorn University Bangkok Thailand; ^2^ Department of Prosthodontics, Faculty of Odontology Malmoe University Malmö Sweden; ^3^ Clinic of Reconstructive Dentistry, Center of Dental Medicine University of Zurich Zurich Switzerland; ^4^ Faculty of Dentistry Universidad Finis Terrae Santiago Chile; ^5^ Department of Oral and Maxillofacial Surgery, Faculty of Dentistry Chulalongkorn University Bangkok Thailand; ^6^ Department of Dental Medicine Karolinska Institute Stockholm Sweden

**Keywords:** dental implants, emergence angle, implant supracrestal complex, peri‐implant tissue

## Abstract

**Objectives:**

The primary aim of this cross‐sectional study was to investigate the association between prosthesis design and peri‐implant mucosa dimensions and morphology. The secondary aim was to investigate associations between mucosal dimensions and the presence of mucositis.

**Materials and Methods:**

Forty‐seven patients with 103 posterior bone level implants underwent clinical and radiographic examination, including cone beam computer tomography and intraoral optical scanning. Three‐dimensional models for each implant and peri‐implant mucosa were constructed. Vertical mucosa height (TH), horizontal mucosa width at implant platform (TW), and 1.5 mm coronal of the platform (TW1.5), as well as mucosal emergence angle (MEA), deep angle (DA), and total contour angle (TA) were measured at six sites for each implant.

**Results:**

There was a consistent correlation between peri‐implant mucosa width and height (*β* = 0.217, *p* < 0.001), with the width consistently surpassing height by a factor of 1.4–2.1. All three angles (MEA, DA, TA) were negatively associated with mucosa height (*p* < 0.001), while DA was negatively associated with mucosa width (TW1.5) (*p* < 0.001, *β* = −0.02, 95% CI: −0.03, −0.01). There was a significant negative association between bleeding on probing (BoP) and mucosa width at platform (OR 0.903, 95% CI: 0.818–0.997, *p* = 0.043) and 1.5 coronal (OR 0.877, 95% CI: 0.778–0.989, *p* = 0.033). Implants with less than half sites positive for BoP (0–2/6) had significantly higher mucosa height (OR 3.51, 95% CI: 1.72–7.14, *p* = 0.001).

**Conclusions:**

Prosthesis design can influence the dimensions of the peri‐implant mucosa, with wider emergence profile angles associated with reduced peri‐implant mucosa height. In particular, a wider deep angle is associated with reduced mucosa width in posterior sites. Reduced peri‐implant mucosa height and width are associated with more signs of inflammation.

**Trial Registration:**

Registered in Thai Clinical Trials Registry: http://www.thaiclinicaltrials.org/show/TCTR20220204002.

## 1 Introduction

Several morphological features and dimensions of the peri‐implant mucosa have been associated with clinical outcomes related to aesthetics, tissue morphology, as well as risk for inflammation. The vertical height of the peri‐implant mucosa has been assessed in humans both in histological studies (Glauser et al. 2005, Romanos et al. 2010, Tomasi et al. 2014) and clinical studies with measurements conducted on tissue impressions (Nozawa et al. 2006, Farronato et al. 2019). The vertical or supracrestal height of the peri‐implant mucosa is a critical anatomic feature, directing the establishment of the critical biological zone of mucosal attachment (Mattheos, Vergoullis, et al. 2021, Monje, González‐Martín, and Ávila‐Ortiz 2023). The horizontal width of the peri‐implant mucosa has also been assessed in clinical studies, albeit commonly by means of lower precision. Traditional assessments of horizontal tissue “thickness” were mainly conducted by assessing the transmucosal visibility of a colored probe (Kan et al. 2003, Evans and Chen 2008, Nisapakultorn et al. 2010), ultrasonic devices (Cardaropoli, Lekholm, and Wennström 2006) or endodontic files (Strauss et al. 2024). More recent studies have employed measurements in three‐dimensional radiographic and optical imaging (Kheur et al. 2015, Khorshed et al. 2023) in a noninvasive, reliable, and reproducible manner (Strauss et al. 2024). The width of the peri‐implant mucosa has been associated with aesthetic outcomes (Bienz et al. 2022), recession (Nozawa et al. 2006, Chen et al. 2009), and papilla fill (Romeo et al. 2008). Interestingly, clinical studies have identified a correlation between peri‐implant mucosa height and horizontal width at the implant platform, with researchers suggesting the width surpasses height by a ratio of 1.5 (Nozawa et al. 2006, Cardaropoli, Lekholm, and Wennström 2006) or 1.19 ± 0.55 (Farronato et al. 2019). Longitudinal studies have further suggested that changes in the height of the mucosa could be regulated by the width (Nozawa et al. 2006, Bengazi, Wennström, and Lekholm 1996). The width‐to‐height ratio of peri‐implant mucosa appears inverted to what was measured on free gingiva around teeth, where the height was found to surpass width by a ratio of 1.5 (Wenstrom et al. 1996) albeit the anatomic location of the measurements in teeth and implants might not be directly comparable. The lack of keratinized mucosa is another morphological feature of the peri‐implant mucosa that has been associated with an increased risk for mucositis (Mancini et al. 2024) and peri‐implantitis (Mahardawi et al. 2023).

Although the dimensions of the peri‐implant mucosa and its relation to clinical outcomes have been investigated in multiple studies, it has scarcely been assessed in parallel to the design of the prosthesis. Siegenthaler et al. (2022) in a prospective study showed that alterations in the contour of the prosthesis could have significant implications for the peri‐implant mucosa height, increasing the occurrence of recession when the contour was switched from concave to convex. The peri‐implant tissue remains a tissue formed as a direct consequence of the implant placement and restoration. Thus its formation, maturation, morphology, and dimensions should be best understood in relation to the conditions that lead to their creation, that is, the surgical placement of the implant‐prosthesis complex (Mattheos, Vergoullis, et al. 2021).

The primary aim of this cross‐sectional study was to investigate associations between peri‐implant mucosa dimensions and morphology (height and width, keratinized zone) with the emergence profile angles of the implant prosthesis (deep angle [DA], mucosal emergence angle [MEA], contour angle [CA]). A secondary aim of the study was to investigate associations between peri‐implant mucosa dimensions, morphology, and the presence of mucositis.

## 2 Materials and Methods

This study was approved by the Ethics Committee at the Faculty of Dentistry, Chulalongkorn University (HREC‐DCU 2022‐024) and was registered at the Thai Clinical Trials Registry (TCTR20210709003). The study followed the STROBE statements.

### 2.1 Patient Sample

Healthy patients (ASA classification I and II) aged 20 years or older, having received implant therapy at the postgraduate clinics of Prosthodontics or the clinic of Implants and Aesthetics, Faculty of Dentistry, Chulalongkorn University and in maintenance for at least 3 months between July 4 and November 30, 2022, were considered for the study, as presented in detail by Rungtanakiat et al. (2023).

Patients were invited to participate in the study if the following conditions were met:

1. Posterior implant supported single crown.
2. Prosthesis axis no more than 10° divergence from implant axis.
3. At least 3 months post restoration.

**Figure 1:**
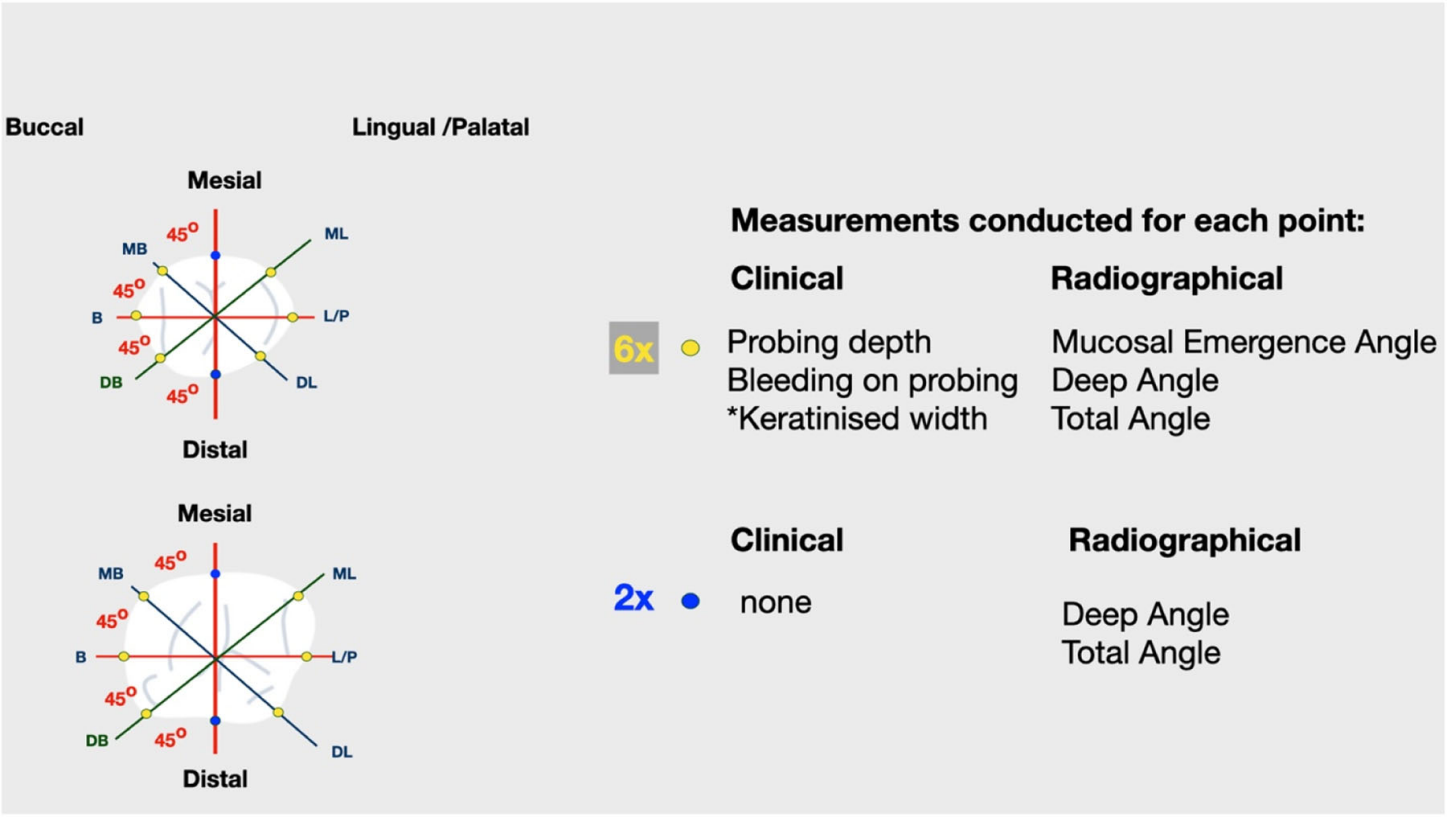
Eight sites (mesial [M], distal [D], mesiobuccal [MB], buccal, distobuccal [DB], and respective lingual/palatal) were defined based on three horizontal planes for each implant crown. Plaque index, bleeding on probing, and probing depth were recorded in six of them (yellow dots), but not for the two directly mesial and distal points (M&D, blue dots). Likewise, mucosal emergence and total and deep angles were calculated for the six sites, but only deep angle was measured for sites M and D. Width of keratinized Mucosa was recorded only at the three buccal sites (MB, B, DB).

Upon informed consent, the patients received clinical and radiographic (cone beam computed tomography [CBCT]) examination (Figure 1), as described in further detail in Rungtanakiat et al. (2023). In the cases where the excessive contour of the prosthesis prevented the insertion of the probe in the reasonably vertical direction, probing depth was not recorded.

### 2.2 Measurements

The DICOM file of CBCT and the intraoral optical scan (STL file) were imported and transposed in the treatment planning software (coDiagnostiX version 9.7, Dental Wings Inc.).

**Figure 2:**
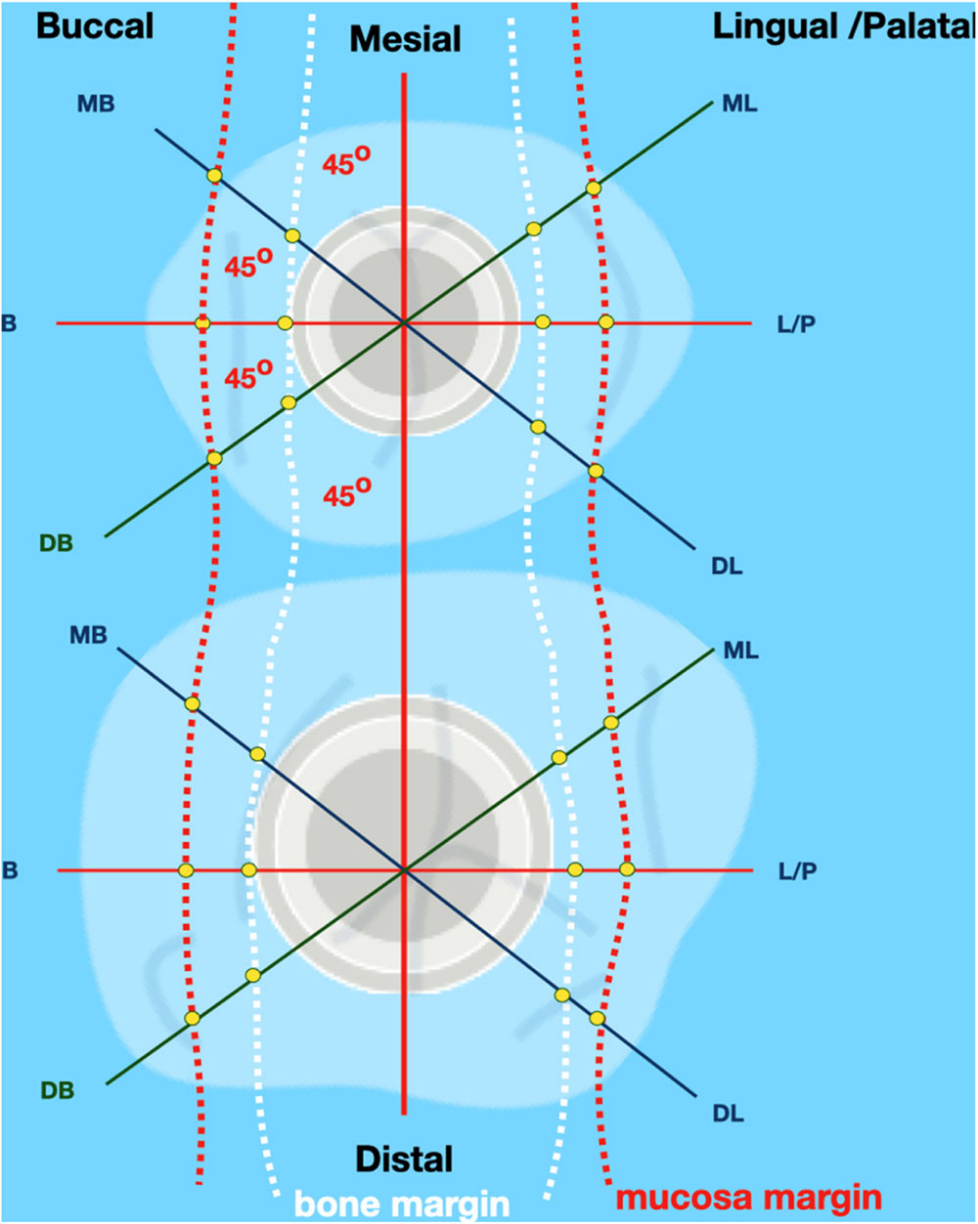
Schematic representation (not in scale) of the horizontal measurements of mucosa dimensions at platform level on the basis of the six defined planes from the bone margin (white dashed line) to the outer margin of the oral mucosa (red dashed line) at six sites per implant.

The implant axis and the perpendicular axis of the implant platform were defined. Thereafter, four axial planes (parallel to the implant axis) were identified (Figure 2):

i. Midline (buccolingual plane).
ii. Interproximal (mesiodistal), perpendicular to the midline plane.
iii. 45° lateral oblique mesiobuccal.
iv. 45° lateral oblique distobuccal.

Each plane included two side views of the Implant Supracrestal Complex, so a total of eight sites were identified for each implant (Figure 1). The following horizontal levels (perpendicular to the implant axis) were defined for each site:

i. Implant platform.
ii. 1.5 mm coronally of the implant platform.
iii. Mucosal margin.
iv. 0.5 mm apically of mucosal margin.

Thereafter, the following points were identified for each level:

A. Point of implant abutment nearest to the implant platform.
B. Point of implant abutment 1.5 mm coronally of point A (projected on implant axis).
C. Most coronal points of soft tissue (mucosal margin).
D. Point of prosthesis 0.5 mm apically of mucosal margin (projected on implant axis).
E. Point of the prosthesis at maximum convexity/prominence.

Three angles were defined as follows:

i. Deep angle (DA): angle of the abutment ascending directly from the implant platform. Defined by the implant axis and the line connecting A–B.
ii. Mucosal emergence angle (MEA): angle of the prosthesis emerging through the soft tissue. Defined by the implant axis and the line connecting C–D.
iii. Total contour angle (CA): angle of the overall contour of the prosthesis. Defined by the implant axis and the line connecting A–E.

**Figure 3:**
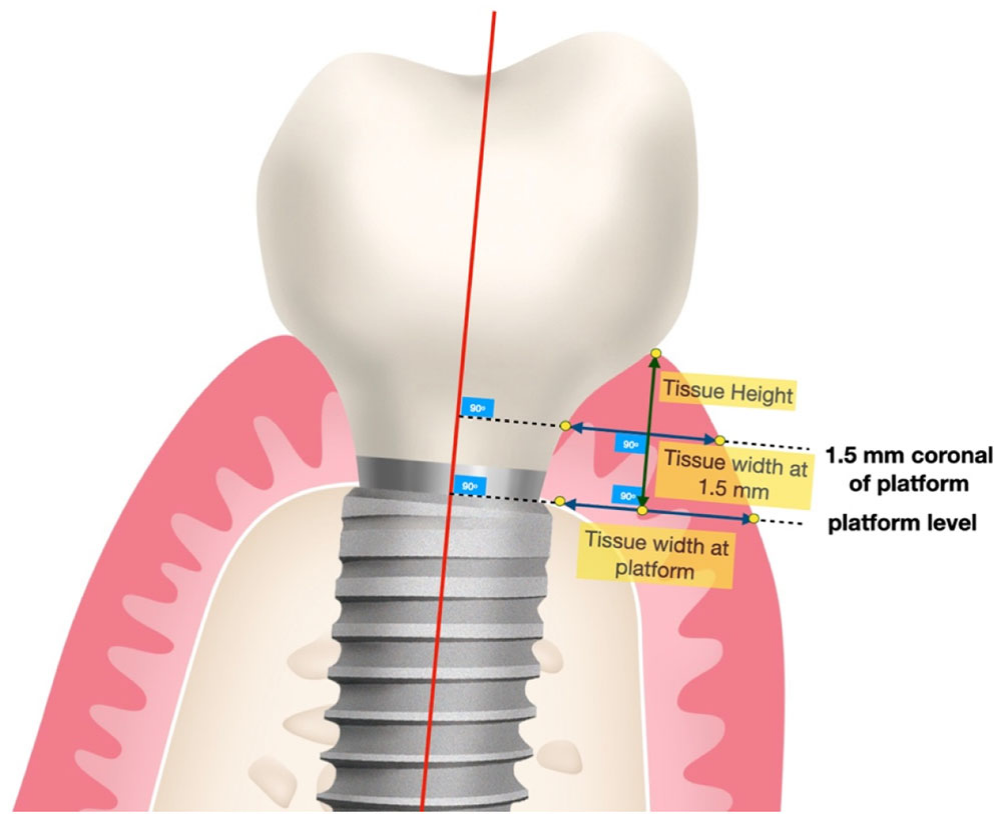
Schematic representation (not in scale) of the measurements conducted for the mucosa height and mucosa width at the platform and 1.5 mm, at six sites per implant.

**Figure 4:**
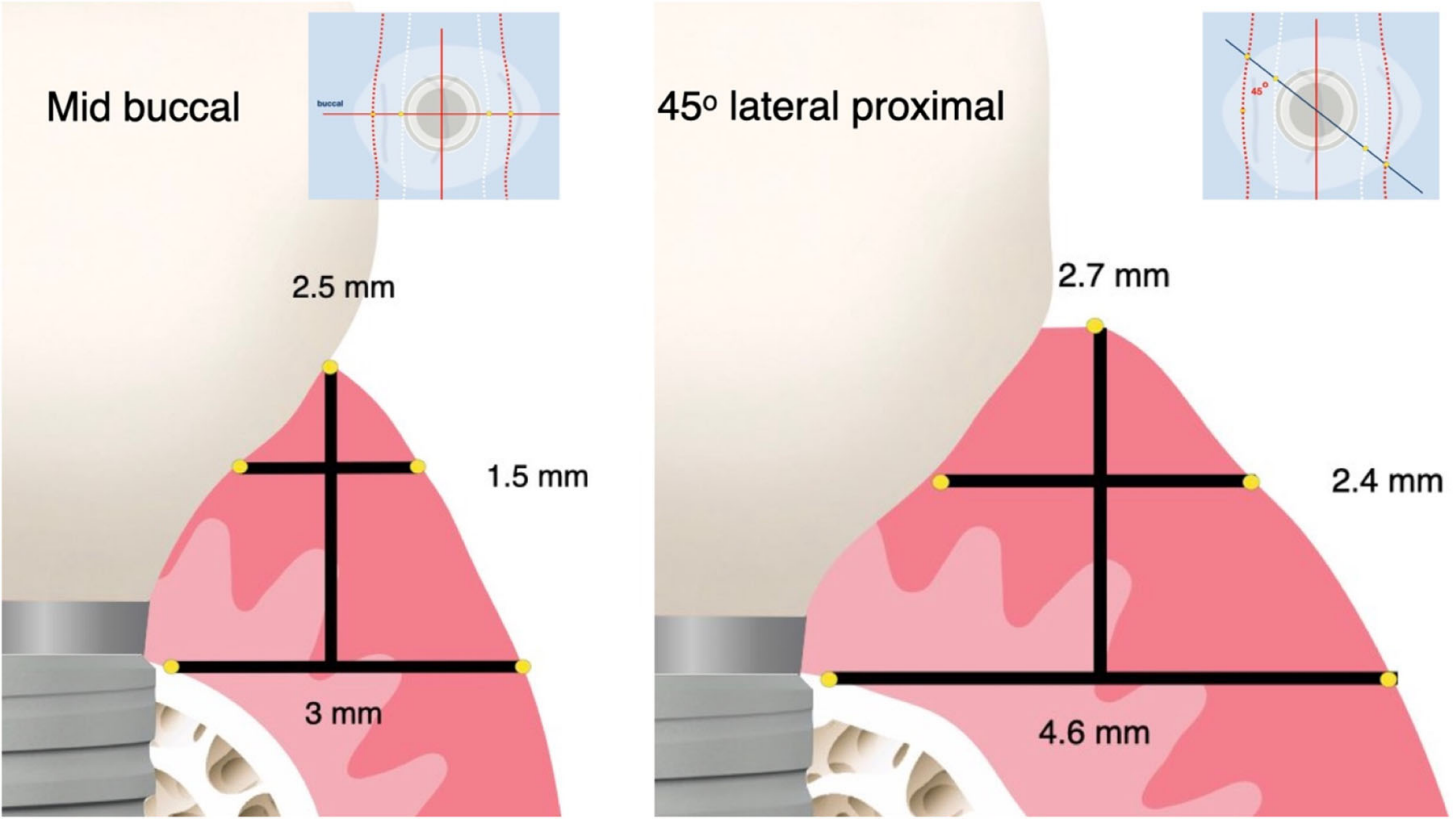
Schematic representation (in scale) of the mucosa dimensions (height, width at platform, and width at 1.5 mm) as directed by the mean of the respective measurement points at mid‐buccal (left) and 45° oblique lateral (right).

Peri‐implant mucosa dimensions were measured as follows (Figures 3 and 4):

i. TwP: Mucosa width at platform. The horizontal dimension of the mucosa is in mm at the implant platform level.
ii. Tw1.5: Mucosa width. The horizontal dimension of the mucosa is in mm, 1.5 mm coronal of the implant platform level.
iii. TH: Mucosa height: The vertical dimension of the mucosa from the implant platform level to the most coronal point of the mucosa, as measured in a perpendicular line (Figures 3 and 4).

All radiographic measurements were conducted by two calibrated examiners, blinded to the clinical examination measurements. Both examiners contributed to the definition of the planes and points of measurement for the study. Thereafter interrater agreement was assessed using a calibration exercise where the two examiners conducted independently a set of 10 measurements. The intraclass correlation coefficient was 0.998 (0.995–0.999), *p* < 0.001.

### 2.3 Clinical Examination

All clinical examinations were conducted by one specialist periodontist and included among others dichotomous registration of the plaque index (O'Leary et al. 1972), dichotomous registration of bleeding on probing (BoP), and presence of suppuration, probing depth rounded to nearest mm at six points per crown with plastic periodontal probe (Hu‐Friedy) and keratinized mucosa width in mm, at three points per crown (Figure 1), as per previously published protocol (Rungtanakiat et al. 2023). Intrarater agreement was assessed by measuring peri‐implant probing depth in four patients at two subsequent visits, 48 h apart. Intraclass correlation coefficient was ICC = 0.931 (95% CI: 0.840−0.970, *p* < 0.001).

### 2.4 Clinical Diagnoses

Diagnosis at the implant level was based on the condition of the peri‐implant tissue upon clinical and radiographical examination. Mucositis was defined as bleeding and/or suppuration on gentle probing and was reported at implant level per site from a total of six sites (0/6–6/6). Three case definitions were used for peri‐implantitis:

a.
  – Bleeding and/or suppuration on gentle probing.
  – Probing depth ≥ 6 mm.
  – Bone loss ≥ 3 mm apical of the most coronal portion of the intra‐osseous part of the implant (day‐to‐day practice, Berglundh et al. 2018).
b.
  – Bleeding and/or suppuration on gentle probing.
  – Bone loss ≥ 3 mm apical of the most coronal portion of the intra‐osseous part of the implant (epidemiological studies, Berglundh et al. 2018).
c.
  – Bleeding and/or suppuration on gentle probing.
  – Bone loss ≥ 2 mm apical of the most coronal portion of the intra‐osseous part of the implant (Romandini et al. 2021).

### 2.5 Statistical Analysis

Descriptive statistics analysis was conducted using mean, standard deviation (SD), median, interquartile range (IQR), and percentages as appropriate. Differences of keratinized mucosa dimensions were analyzed using a one‐way repeated measures analysis of variance (ANOVA) while differences between sites with a one‐way ANOVA. Differences in mucosal height and width and exploration of the associations between prosthetic angles and tissue dimensions or conditions were investigated using a linear mixed model and adjustment for the site as a fixed factor. Patient level and implant level were random intercepts and random slope of implant level on site. A logistic mixed model was performed to explore the association between tissue width, tissue height, and the presence of BOP, adjusting for sites and patient level as a random intercept. Statistical testing was done within an exploratory framework at a two‐sided significance level of *α* = 0.05. No correction of the multiple testing was applied. The nature of this study was exploratory and no sample size calculation was conducted. All statistical tests were performed using Stata IC15 (StataCorp, 2017, College Station, TX, USA).

## 3 Results

Measurements from 47 patients (24 male and 23 female) with 103 bone level implant crowns in molar and premolar positions were analyzed. Patients' average age was 61 years (range: 32–81) and the average time with prosthesis in function was 39 months (3 years, 3 months) (SD 28.05 months, range 3–120 months). Eighty‐eight crowns were screw‐retained (85.5%) and 15 were cement‐retained (14.5%), with the screw‐retained being splited almost equally to porcelain fused to metal (*n* = 42) and monolithic Zirconia on Ti‐base abutment (*n* = 45).

### 3.1 Mean Tissue Dimensions

The mucosal height ranged from 2.1 to 3.3 mm with the mean value at all six sites between 2.5 and 2.7 mm. The lateral sites presented with somewhat higher values than the midbuccal/lingual sites, without this difference reaching statistical significance (Table 1). The trend for vertical mucosal height was to increase from first premolar to second molar. Mean mucosal width at platform ranged from 2.1 to 5.6 mm, with the mean values between 3.0 and 4.6 mm at the six measurement points. The mucosa at lateral lingual/palatal measuring points was significantly thicker than the respective midbuccal/lingual. There was a clear trend for the mucosal width to increase at the platform from first premolar to second molar, which was statistically significant for three out of six sites. At 1.5 mm higher than the platform the mean width decreased by an average of 52% to 1.5–2.4 mm. Again, there was a trend for the mucosal width to increase at this level from first premolar to second molar, which reached statistical significance at one of the six sites.

**Table 1:** Mean and standard deviation of peri‐implant mucosa dimensions for first (x4), second (x5) premolar, and respective molar (x6, x7) sites.

	Implant site				
	x4 (*N* = 13)	x5 (*N* = 19)	x6 (*N* = 54)	x7 (*N* = 17)	*p* value between position^a^
TP BM	3.1 (1.3)	3.0 (1.1)	3.7 (1.8)	4.6 (1.7)	0.147
TP BB	2.1 (1.1)	2.9 (1.3)	2.8 (1.2)	4.6 (1.7)	0.040
TP BD	2.9 (1.2)	3.4 (1.4)	4.2 (2.1)	5.6 (2.4)	0.206
TP LM	4.8 (2.9)	4.3 (3.4)	4.7 (3.3)	4.4 (2.5)	0.001
TP LL	3.7 (2.7)	3.6 (2.6)	3.2 (1.9)	3.9 (2.9)	0.192
TP LD	4.1 (2.9)	4.2 (2.6)	4.3 (3.6)	4.1 (2.6)	0.045
*p* value between sites^a^	0.001	0.810	0.221	< 0.001	
T1.5 BM	1.5 (1.2)	1.6 (1.2)	2.0 (1.6)	2.4 (1.4)	0.679
T1.5 BB	1.2 (0.8)	1.4 (1.4)	1.3 (1.1)	2.7 (1.4)	0.124
T1.5 BD	1.4 (0.9)	1.6 (1.7)	2.0 (1.7)	3.2 (2.2)	0.642
T1.5 LM	2.6 (2.8)	2.2 (2.9)	2.5 (2.7)	2.4 (1.9)	0.0.50
T1.5 LL	1.7 (2.2)	1.8 (2.4)	1.6 (1.5)	2.2 (2.1)	0.509
T1.5 LD	2.1 (2.6)	2.3 (2.1)	2.5 (3.2)	2.3 (2.0)	0.143
*p* value between sites^a^	0.103	0.977	0.812	0.058	
TH BM	2.6 (1.2)	2.5 (1.2)	2.5 (1.1)	3.0 (1.0)	0.471
TH BB	2.2 (1.0)	2.3 (1.2)	2.4 (1.0)	3.3 (1.2)	0.406
TH BD	2.6 (1.0)	2.4 (1.4)	2.5 (1.2)	3.2 (1.5)	0.960
TH LM	2.8 (0.9)	2.6 (1.5)	2.8 (1.4)	2.9 (1.0)	0.394
TH LL	2.3 (1.1)	2.6 (1.4)	2.3 (1.1)	2.8 (1.3)	0.522
TH LD	2.1 (1.0)	2.8 (1.5)	2.6 (1.4)	3.0 (1.5)	0.389
*p* value between sites^a^	0.192	0.304	0.655	0.161	

Abbreviations: BM, BB, BD = bucco‐mesial, ‐midbuccal, and ‐distal; LM, LL, LD = lingual‐mesial, ‐midlingual, and ‐distal; T1.5 = tissue width at 1.5 mm; TH = vertical tissue height; TP = tissue width at the platform.

^a^ *p* values for the site and position effects were estimated using a linear mixed model, including patient and implant levels as a random effect.

### 3.2 Height/Width Ratio

The mucosa width/height ratio collectively for all measurement points ranged from 1.4 to 2.1 for the width at the platform and 0.6–0.8 for the width at 1.5 mm (Table 2). There was no association between the width/height ratio and bleeding on probing or any of the case definitions for the clinical diagnosis of peri‐implantitis.

**Table 2:** Mean peri‐implant mucosa width/height ratio and standard deviation for width at platform and 1.5 mm. Mean per respective implant site first (x4), second (x5) premolar and first (x6) and second molar (x7).

	Implant site				
Width/height	x4 (*N* = 13)	x5 (*N* = 19)	x6 (*N* = 54)	x7 (*N* = 17)	*p* value^a^
At implant platform					
BM	1.4 (0.9)	1.3 (0.4)	1.7 (0.9)	1.5 (0.6)	0.719
BB	1.5 (2.1)	1.4 (0.5)	1.4 (1.4)	1.4 (0.6)	0.728
BD	1.2 (0.7)	2.1 (2.6)	2.4 (2.8)	1.9 (0.6)	0.007
LM	1.8 (1.0)	1.5 (0.7)	1.9 (1.4)	1.7 (1.0)	0.741
LL	1.7 (1.0)	1.5 (1.0)	1.5 (0.9)	1.4 (0.9)	0.869
LD	1.9 (0.8)	1.5 (0.6)	1.7 (1.0)	1.4 (0.8)	0.823
*p* value between sites^a^	0.715	0.450	0.113	0.662	
At 1.5 mm platform					
BM	0.5 (0.3)	0.6 (0.4)	0.7 (0.6)	0.8 (0.5)	0.655
BB	0.8 (1.3)	0.5 (0.5)	0.5 (0.3)	0.8 (0.4)	0.049
BD	0.5 (0.3)	0.7 (0.8)	0.7 (0.6)	0.9 (0.6)	0.957
LM	0.8 (0.9)	0.7 (0.6)	0.8 (0.7)	0.8 (0.4)	0.391
LL	0.6 (0.8)	0.5 (0.4)	0.6 (0.4)	0.7 (0.5)	0.345
LD	0.8 (0.8)	0.7 (0.5)	0.7 (0.8)	0.7 (0.5)	0.491
*p* value between sites^a^	0.244	0.577	0.123	0.334	

^a^ *p* values for the site and position effects were estimated using a linear mixed model, including patient and implant levels as a random effect.

### 3.3 Differences at Molar/Premolar Sites and Maxillary/Mandibular Sites

When molars and premolars were analyzed separately, the mucosal width at the platform was significantly greater for three of the six sites of molars, and two out of six at 1.5 mm (Table 3). There was no significant difference in mucosa height between premolar and molar sites.

**Table 3:** Mean and standard deviation of peri‐implant mucosa dimensions for implants at premolar and molar sites.

	Premolars (x4, x5) (*N* = 32)	Molars (x6, x7) (*N* = 71)	*p* value^a^
At implant platform			
BM	3.1 (1.2)	3.9 (1.8)	0.137
BB	2.6 (1.3)	3.2 (1.6)	0.031
BD	3.2 (1.3)	4.6 (2.2)	0.102
LM	4.5 (3.2)	4.6 (3.1)	< 0.001
LL	3.7 (2.6)	3.4 (2.2)	0.054
LD	4.2 (2.7)	4.2 (3.4)	0.026
*p* value between sites^a^	< 0.001	0.024	
At 1.5 mm platform			
BM	1.6 (1.2)	2.1 (1.6)	0.400
BB	1.3 (1.2)	1.7 (1.3)	0.115
BD	1.5 (1.4)	2.3 (1.9)	0.693
LM	2.4 (2.8)	2.5 (2.5)	0.021
LL	1.8 (2.3)	1.7 (1.6)	0.207
LD	2.2 (2.3)	2.5 (3.0)	0.066
*p* value between sites^a^	0.013	0.459	
Tissue height			
BM	2.5 (1.2)	2.6 (1.1)	0.945
BB	2.3 (1.1)	2.6 (1.1)	0.468
BD	2.5 (1.2)	2.6 (1.3)	0.981
LM	2.7 (1.3)	2.8 (1.3)	0.348
LL	2.5 (1.3)	2.5 (1.2)	0.374
LD	2.5 (1.3)	2.7 (1.4)	0.953
*p* value between sites^a^	0.500	0.843	

^a^ *p* values for the site and position effects were estimated using a linear mixed model, including patient and implant levels as a random effect.

The mucosal width was significantly more at maxillary than mandibular corresponding sites, but the difference was less pronounced with regard to mucosal height, which reached significance for only one of the six sites (Table 4).

**Table 4:** Mean and standard deviation of peri‐implant mucosa dimensions for implants at mandibular and maxillary sites.

	Maxillary premolars (*N* = 10)	Mandibular premolars (*N* = 22)	Maxillary molars (*N* = 13)	Mandibular molars (*N* = 58)	*p* value between position^a^
TP BM	3.7 (1.0)	2.8 (1.1)	5.5 (1.8)	3.6 (1.7)	0.007
TP BB	2.8 (0.9)	2.5 (1.4)	3.0 (1.2)	3.3 (1.6)	< 0.001
TP BD	3.9 (1.1)	2.9 (1.3)	4.0 (1.9)	4.7 (2.3)	< 0.001
TP LM	6.7 (1.8)	3.5 (3.2)	8.1 (4.1)	3.8 (2.2)	< 0.001
TP LL	5.0 (1.3)	3.1 (2.8)	5.8 (2.5)	2.8 (1.7)	0.029
TP LD	5.6 (2.5)	3.5 (2.5)	8.2 (5.1)	3.3 (2.0)	< 0.001
*p* value between sites^a^	< 0.001	0.001	0.081	< 0.001	
T1.5 BM	1.9 (0.9)	1.4 (1.3)	3.2 (1.8)	1.9 (1.4)	0.128
T1.5 BB	1.5 (0.6)	1.2 (1.4)	1.3 (1.1)	1.7 (1.3)	0.003
T1.5 BD	2.0 (1.1)	1.3 (1.5)	1.9 (1.9)	2.4 (1.9)	0.017
T1.5 LM	4.0 (1.4)	1.6 (3.0)	5.1 (3.9)	1.9 (1.7)	< 0.001
T1.5 LL	2.2 (1.2)	1.6 (2.6)	3.1 (2.2)	1.4 (1.3)	0.379
T1.5 LD	3.5 (1.9)	1.7 (2.2)	5.4 (5.1)	1.8 (1.7)	< 0.001
*p* value between sites^a^	< 0.001	0.004	0.040	< 0.001	
TH BM	2.6 (0.9)	2.5 (1.3)	2.8 (0.8)	2.6 (1.2)	0.688
TH BB	2.3 (1.1)	2.3 (1.1)	2.4 (1.1)	2.7 (1.1)	0.478
TH BD	2.7 (1.0)	2.4 (1.3)	2.1 (1.0)	2.8 (1.3)	0.137
TH LM	3.6 (1.0)	2.3 (1.3)	3.6 (1.6)	2.6 (1.2)	0.001
TH LL	3.0 (0.9)	2.2 (1.4)	3.0 (1.1)	2.3 (1.2)	0.126
TH LD	3.1 (1.1)	2.3 (1.4)	3.1 (1.7)	2.6 (1.3)	0.367
*p* value between sites^a^	0.002	< 0.001	0.406	0.001	

Abbreviations: BM, BB, BD = bucco‐mesial, ‐midbuccal, and ‐distal; LM, LL, LD = lingual‐mesial, ‐midlingual, and ‐distal; T1.5 = tissue width at 1.5 mm; TH = vertical tissue height; TP = tissue width at the platform.

^a^ *p* values for the site and position effects were estimated using a linear mixed model, including patient and implant levels as a random effect.

### 3.4 Association of Tissue Height and Width

There was a positive correlation between mucosal width at the platform and mucosal vertical height (*β* = 0.217, *p* < 0.001) (Table 5). There was also a strong correlation between tissue width at the platform and tissue width at 1.5 mm (*β* = 1.06, *p* < 0.001). It is noteworthy that the average mucosal width at 1.5 mm coronally decreases to only 52% of the width at the platform, with this width being around 50% for premolars, 51% for the first and 55% for the second molar. At the same time, the mucosal width increases on average from 3.25 mm mid‐buccal/lingual to 4.15 at oblique lateral mesio/distal at platform (127% increase) and from 1.6 mm to 2.225 mm at 1.5 mm (139% increase).

**Table 5:** Unstandardized coefficient (*β*) and confidence intervals (CI) between mucosa height and deep angle (DA), mucosal emergence angle (MEA), total contour angle (TA), mucosa width (*n* = 618). Linear mixed model and adjustment for the site as a fixed factor. Patient level and implant level are random intercepts and random slope of implant level on site.

	*β* [95% CI]	*p* value
Tissue width at the platform	0.217	< 0.001
	[0.185, 0.248]	
Tissue width at 1.5 mm above the platform	0.323	< 0.001
	[0.286, 0.360]	
Deep angle	−0.018	< 0.001
	[−0.024, −0.011]	
Total contour angle	−0.015	< 0.001
	[−0.021, −0.008]	
Mucosal emergence angle	−0.011	< 0.001
	[−0.015, −0.006]	

### 3.5 Mucositis/Peri‐Implantitis

Mucositis at the implant level was assessed by the number of bleeding sites out of a total of six (Vianna et al. 2018), as seen in Rungtanakiat et al. (2023). When divided into two groups, implants with 0–2/6 bleeding sites were 37 (37%) while those with half or more bleeding sites out of six were 66 (63%) (Table 6). The prevalence of peri‐implantitis for the three case definitions used (a: PD ≥ 6 and BL ≥ 3, b: BL ≥ 3 mm, and c: BL ≥ 2 mm was 2 (1.9%), 8 (7.8%), and 10 (9.7%), respectively (Rungtanakiat et al. 2023).

**Table 6:** Presence of positive sites for bleeding on probing (BoP), organized according to the number of bleeding on probing sites per implant (Rungtanakiat et al. 2023).

		Implants (*n* = 103)
Bleeding on probing	BoP+, 0–2 of 6 sites	37 (35.92%)
	BoP+, 3 of 6 sites	27 (26.21%)
	BoP+, 4 of 6 sites	14 (13.59%)
	BoP+, 5 of 6 sites	13 (12.62%)
	BoP+, 6 of 6 sites	12 (11.65%)

### 3.6 Correlation Between Prosthetic Angles and Tissue Dimensions

**Figure 5:**
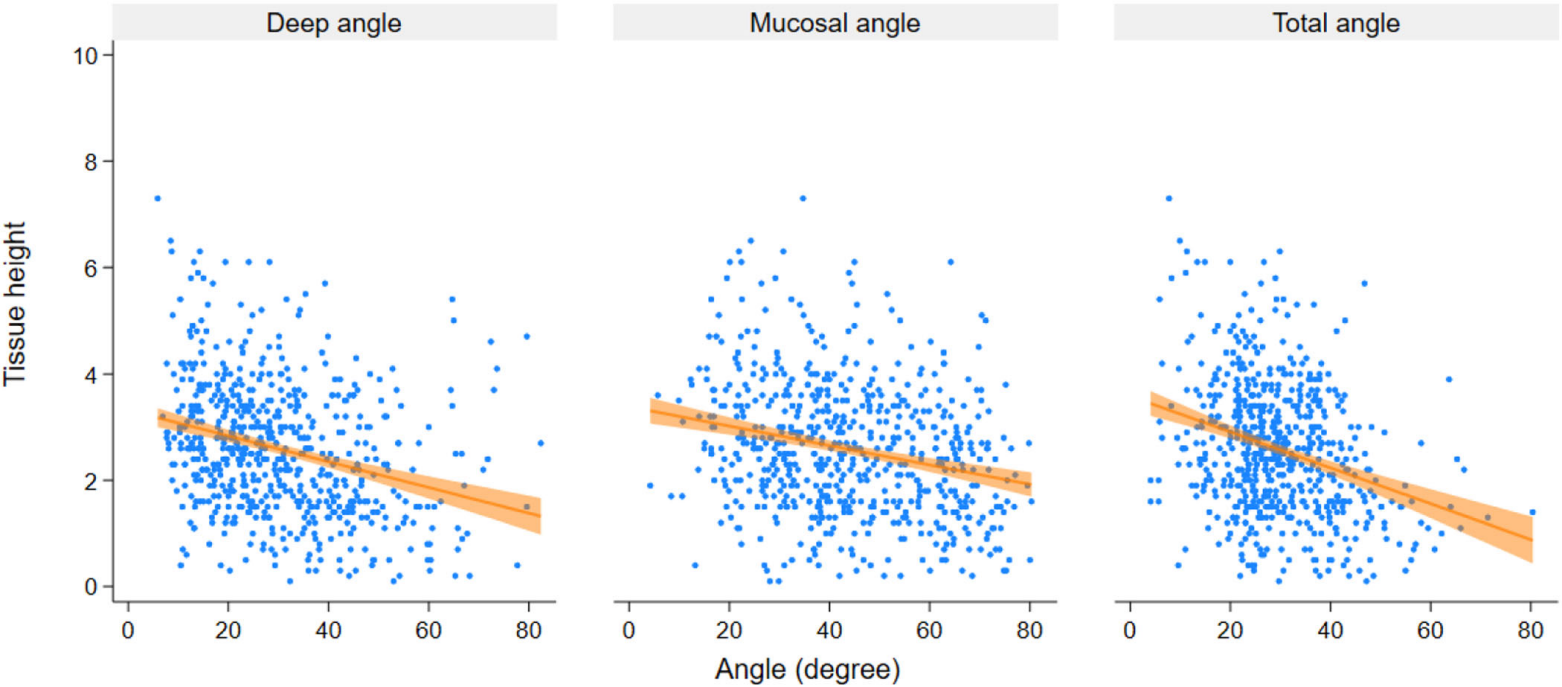
Correlation between prosthetic angles and vertical mucosa height. Linear mixed model and adjustment for the site as a fixed factor. Patient level and implant level are random intercepts and random slope of implant level on site.

The adjusted linear mixed model revealed that all prosthetic angles (deep angle, total contour angle, and mucosal emergence angle) were negatively associated with vertical mucosal height (*p* < 0.001, Table 5, Figure 5). Expressed differently, the increase in any of the aforementioned angles was significantly associated with a decrease in soft tissue height.

**Figure 6:**
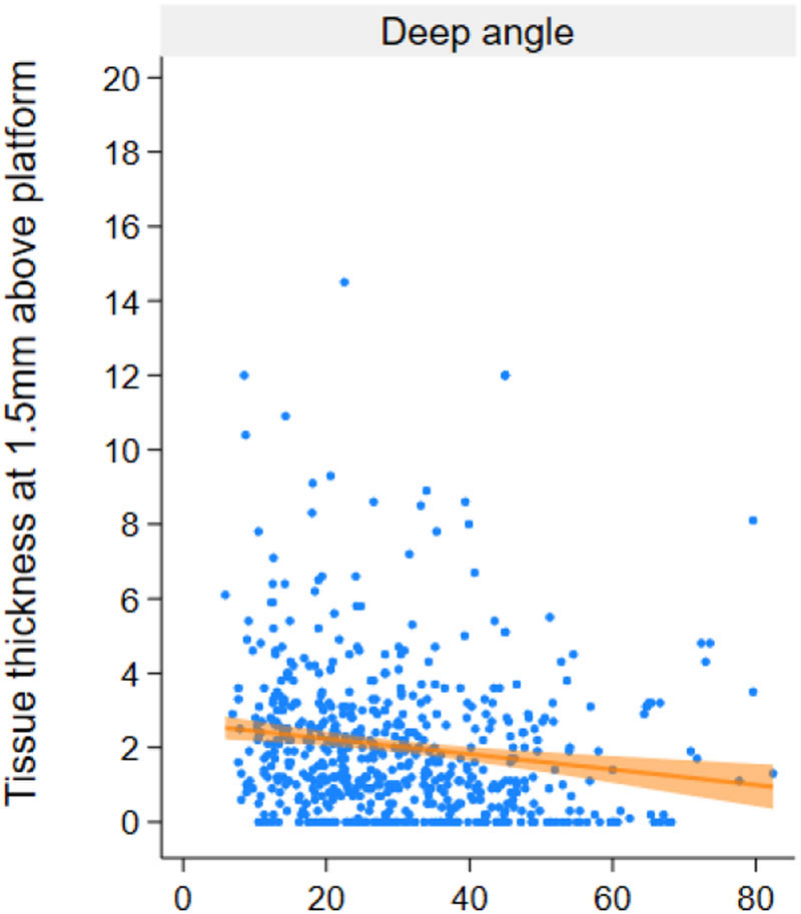
Correlation between deep angle (DA) and width at 1.5 mm coronal of the platform. Linear mixed model and adjustment for the site as a fixed factor. Patient level and implant level are random intercepts and random slope of implant level on site.

There was also a negative correlation between deep angle and tissue width at 1.5 mm (*p* < 0.001, *β* = −0.02, 95% CI: −0.03, −0.01) (Figure 6). The deep angle had a significant positive correlation with the ratio of tissue width/tissue height—at the platform (*β* = 0.36, 95% CI: 0.03–0.04), but not at the 1.5 mm height.

### 3.7 Correlation Between Tissue Dimensions and Peri‐Implantitis/Mucositis

There was no correlation between mucosa dimensions and diagnosis of peri‐implantitis, but there was a significant negative association between mucosal width and dichotomous bleeding on probing, with an odds ratio of 0.903 (95% CI: 0.818–0.997, *p* = 0.043) for width at platform level and 0.877 at 1.5 mm level (95% CI: 0.778–0.989, *p* = 0.033). This indicates that an increase in the mucosa width reduced the odds of presenting bleeding on probing.

Furthermore, implants presented with no more than two bleeding sites out of the six assessed had significantly higher mucosa height than implants with three or more sites found to be bleeding on probing (OR 3.51, 95% confidence interval [1.72–7.14], *p* = 0.001).

### 3.8 Keratinized Mucosa

The mean keratinized tissue width at buccal sites (KBM, KBB, KBD) was 2.6 ± 1.6, 2.0 ± 1.5, and 2.4 ± 1.7 mm, respectively (Table 7) and it was significantly wider at lateral oblique points of measurement than midbuccal. Also, it was significantly higher at the first premolar with a trend to decrease toward the molars and being the lowest at the second molar. An association of keratinized mucosa and tissue height only reached the level of significance for the buccal‐mesial sites. None of the implant sites presented with 0 width of keratinized mucosa at all three buccal sites, 12 implants had no keratinized mucosa at two of the three sites while 12 implants at one site, respectively, with the mid‐buccal point most likely to present with no keratinized mucosa (19 implants). No association was found between the width of keratinized mucosa and bleeding on probing for the corresponding sites.

**Table 7:** Mean keratinized mucosa dimensions (mm) and standard deviation for each of the three buccal sites were measured.

		Position				
Keratinized mucosa width	Total (*N* = 103)	x4 (*N* = 13)	x5 (*N* = 19)	x6 (*N* = 54)	x7 (*N* = 17)	*p* value between position^b^
BM	2.6 (1.6)	4.0 (1.8)	2.7 (1.2)	2.5 (1.5)	1.7 (1.2)	< 0.001
BB	2.0 (1.5)	3.3 (1.7)	1.7 (1.3)	2.1 (1.5)	1.2 (1.3)	0.001
BD	2.4 (1.7)	4.0 (1.6)	2.4 (1.4)	2.4 (1.6)	1.1 (1.2)	< 0.001
*p* value between sites^a^	< 0.001	0.006	< 0.001	0.005	0.003	

Abbreviations: BM, BB, BD = bucco‐mesial, ‐midbuccal, and ‐distal; x4 = first premolar, x5 = second premolar, x6 = first molar, x7 = second molar.

^a^ One‐way repeated ANOVA.

^b^ One‐way ANOVA.

## 4 Discussion

This cross‐sectional study aimed to describe and explore associations between the design of implant‐supported prosthesis and peri‐implant mucosa dimensions in a sample of posterior platform switching implants in patients under maintenance. To achieve this, the study utilized reproducible measurements in a three‐dimensional imaging model based on specific planes and angles, as previously described by Rungtanakiat et al. (2023). This study predominantly revealed:

− A consistent correlation between peri‐implant mucosa width and height.
− A significant association between the design features of the implant‐supported prosthesis (deep angle, mucosal emergence angle, and total contour angles) and the dimensions of the peri‐implant mucosa.

The presence of a width/height ratio in the peri‐implant mucosa is something that has been previously reported in clinical studies, albeit with smaller and less homogenous samples. Nozawa et al. analyzed the volume of soft tissue around internal hexagon single implants with a flat‐to‐flat connection on 14 patients after an average period of 3 years and 5 months. The ratio between the height and width of peri‐implant mucosa was 1.58. Farronato et al. (2019) studied mucosa width/height on 20 patients with 32 bone level, platform switching implants and reported a significant association between width and height and a ratio of 1.19 ± 0.55. This trend was confirmed by the present data as well, as in all cases the width of the peri‐implant mucosa at the platform was higher than the vertical mucosa height in the corresponding site. Nevertheless, the results of this study add two new important parameters: First, the ratio between width and height presents with variation between anatomic locations and can vary even within a relatively limited group, such as posterior premolars and molars. Second, peri‐implant tissue dimensions as well as the ratio of width/height can vary depending on the anatomic location within the same implant site. As previous research has been conducted using two‐dimensional imaging, tissue measurements have been mainly focused in the mid‐buccal and at times mid‐palatal points, which in this study are shown to be the sites with the lowest values in terms of both height and width of the mucosa. The thickness of the tissue at the mesial and distal lateral measurement points is significantly more than at the mid‐buccal point, not only at the platform level but also 1.5 mm higher, where the abutment/prosthesis inevitably widens. This “geometrical” bias induced by the overreliance on two‐dimensional imaging for understanding a three‐dimensional structure such as the Implant Supracrestal Complex, might in the past have influenced our understanding and definitions of tissue “thickness.” In the future, it might be essential to review and expand our definitions of peri‐implant tissue phenotype and thickness, to better represent the three‐dimensional complexity of the peri‐implant mucosa taking advantage of new imaging technologies (Strauss et al. 2024).

The same might be true for the width of the keratinized mucosa, which was the lowest at the midbuccal point of measurement. Nevertheless, even if the mid‐buccal point was the site most likely to have no keratinized mucosa (18% of implants), there were no implants in this sample that had no keratinized mucosa at all three measured points. This might explain why the keratinized mucosa width had no impact on bleeding on probing in the sample. Indeed, a recent 10‐year prospective study revealed that the absence of buccal keratinized mucosa around implants was associated with higher risks of presenting buccal bleeding on probing (Mancici et al. 2024). However, the association between a lack of keratinized mucosa and bleeding on probing seems weak (Ravidà et al. 2022). Future studies might need to look closer into the importance of the three‐dimensional arrangement of the keratinized mucosa around implants as most studies have only evaluated the keratinized mucosa at midfacial buccal sites, neglecting mesial and distal sites as well as lingual sites.

Past studies have observed that the width‐to‐height ratio in the case of free gingiva around natural teeth is inverted to this around implants, showing height surpassing width by a ratio of 1.5 (Wennström 1996). Such measurements, taken at the margin of free gingiva, might not be anatomically directly comparable with those in the peri‐implant mucosa and can only serve as a reminder of the important structural differences between two fundamentally different tissues. The clinical significance of the width‐to‐height ratio might not be at its absolute value. The results of this study suggest an underlying geometrical architecture of the peri‐implant mucosa, with the essential establishment of a wide base to support the essential tissue height. In the case of the peri‐implant mucosa, this base might have to be much wider than in physiological periodontal tissue. The width of the peri‐implant mucosa at the implant site depends on many factors, such as the local site anatomic conditions and the position of the implant. However, the results of this study suggest that the prosthesis design might be another important determinant of the peri‐implant mucosa dimensions. In particular, the association of the deep angle with mucosal width is something that has not been presented before. From a purely geometrical point of view, the peri‐implant mucosa appears to fill a pyramid shape, supplementary to the trapezoid shape of the implant prosthesis, when the prosthetic contour remains within 30°, as recommended by Puisys et al. (2023). The deep angle has a critical importance for the marginal bone, as studies have shown increased marginal bone remodeling (Valente et al. 2020) and early marginal bone loss when this angle exceeds 28°–34° (Han et al. 2023). Although defining a cut‐off point has not been attempted in this study and might not be meaningful in clinical terms, the clear negative correlation of the deep angle with the width of the peri‐implant mucosa could suggest further implications of this critical design feature extending beyond marginal bone remodeling. The relation of the prosthetic angles with the peri‐implant mucosa dimensions might come as no surprise to anyone who has been conducting post‐implant placement “tissue conditioning” using prospective contour modifications of provisional prosthesis (Furze et al. 2019). Thus, the results of this study might be well aligned with tissue manipulation techniques such as “dynamic compression” (Wittneben et al. 2013), where contour adjustments of a provisional crown are used to invoke changes in the dimensions and morphology of the peri‐implant mucosa. Furthermore, these results could indirectly explain clinical outcomes such as the increased recession observed when switching from concave to convex contour in anterior implant crowns (Siegenthaler et al. 2022).

Two more associations emerged as the results of this study with regard to clinical outcomes: First, mucosal width was negatively associated with bleeding on probing, especially in the 1.5 mm level coronally of the platform, but not mucosal height. When the implant sites were further stratified in those with no or relatively few bleeding points (0–2/6) and those with half or more of the measured points showing bleeding (3–6/6), there was a significant association of the first with higher vertical tissue height. Such results should be interpreted with caution in a cross‐sectional study since causality cannot be determined. Therefore, they should be seen as preliminary findings that warrant validation through longitudinal studies. Nevertheless, these observations might be well aligned with current research understanding and hint toward wider observed associations (Mattheos, Janda, et al. 2021). With regard to mucosal width, cross‐sectional studies have suggested an increased prevalence of mucositis in the presence of “thin” peri‐implant mucosa (Gharpure et al. 2021, Tur and Sarıbaş 2023). In fact, soft tissue augmentation at implant sites has been advocated as an intervention to promote peri‐implant health over time (Tavelli et al. 2021, Thoma et al. 2018). Conversely, other prospective clinical studies have failed to find any association between phenotype and mucositis (Fernandes‐Costa et al. 2019). This contrast results might be explained by the case definition of mucositis at the implant level. For example, bleeding on probing could differ between thin and thick mucosa because thin mucosa might be more prone to trauma during probing, while the contour of the prosthesis might also interfere with our ability to probe and the consequent registration of bleeding and probing depth (Rungtanakiat et al. 2023). The 2017 World Workshop defined mucositis as the presence of bleeding and/or pus on gentle probing, with or without increased probing depth compared to previous exams, and no bone loss beyond initial remodeling changes (Berglundh et al. 2018). The bleeding on probing was in addition characterized as “profuse” (line or drop, not “dot”) (Renvert et al. 2018), to distinguish inflammation from potential traumatic bleeding, especially when dichotomous scoring is used. Other case definitions proposed later, appeared to focus on dichotomous bleeding on probing in isolation of other signs and qualitative features, where mucositis was defined as probing resulting in two or more bleeding “dots” after probing six sites around an implant (Herrera et al. 2023). Nevertheless, even with consistent force the prevalence of bleeding on probing is influenced by many factors (Dukka et al. 2021), such as the operator and the probing force (Gerber et al. 2009), the morphology and characteristics of the tissue (Ravidà et al. 2022), and the contour of the prosthesis (Rungtanakiat et al. 2023). There is a high likelihood of false positives when relying on dichotomous scoring of bleeding. Common sense would suggest that if one bleeding “dot” can be attributed to traumatic probing, then a second one should be at least as likely in the same implant under the same conditions. Two traumatic “dots” would not equal one inflammatory. Thus the authors have chosen to report the frequency of bleeding sites out of six per implant, allowing for wider interpretations of the dichotomous bleeding data. In the future, case definitions of mucositis based on qualitative features of bleeding on probing in combination with other signs of inflammation (Dukka et al. 2021) might offer a better diagnostic potential to epidemiological studies.

The variation in mucosal height in this sample of posterior implants was rather narrow, with the great majority of sites falling within 2.4–2.8 mm. Implants with peri‐implant mucosa toward the upper margin of this range were associated with significantly fewer bleeding sites. An optimal range of mucosa height facilitating peri‐implant tissue health cannot be easily defined based on a cross‐sectional study, it is however reasonable to expect that an adequate soft tissue height may enhance the structural seal around the implant, potentially better coping with the bacterial challenge and facilitating oral hygiene measures. Furthermore, a larger soft tissue height may relate to the presence of attached (non‐mobile) mucosa (Tarnow et al. 2021). Together, these factors emphasize the importance of the establishment of the essential vertical attachment zone (Mattheos, Janda, et al. 2021). However, this may be influenced by other factors, such as the mucosal emergence angle. A wider angle has been linked to both a higher prevalence of mucositis and a reduced mucosal height.

The major strength of the current study is the detailed and reproducible measurements, defined by points on specific planes, following the model of analysis of cephalometric radiographs. Furthermore, although no specific attempt was made to assess the appropriateness of the implant position, restorations were excluded when the axis of the crown differed from that of the implant by more than 10°. Thus, the great majority of the implant crowns were screw‐retained. Nevertheless, this method also has limitations. Although four axial planes were defined leading to eight distinct sites for each implant, the peri‐implant mucosa margin was only visible in six of them. The outline of the peri‐implant mucosa was not visible directly mesial and distal (under the contact point), the point which was also not accessible to clinical examination. Thus values for MEA, bleeding on probing, probing depth, and plaque score were not recorded for these points, while values for deep angle and total contour angle were not included in the analysis. Furthermore, one should interpret the results of this study in light of the limitations of the sample, which albeit rather homogenous, was collected from the patient pool of one university department.

## Author Contributions

**Piboon Rungtanakiat:** data curation, formal analysis, investigation, writing – original draft preparation. **Natchaya Thitaphanich:** data curation, investigation. writing – review and editing. **Martin Janda:** conceptualization, methodology, validation, visualization, writing – review and editing. **Franz Josef Strauss:** validation, writing – review and editing. **Mansuang Arksornnukit:** conceptualization, formal analysis, funding acquisition, methodology, project administration, resources, software, supervision, validation, writing – review and editing. **Nikos Mattheos:** conceptualization, formal analysis, methodology, project administration, resources, software, supervision, validation, visualization, writing – original draft preparation, writing – review and editing.

## Conflicts of Interest

The authors declare no conflicts of interest.

## Data Availability

The data that support the findings of this study are available from the corresponding author upon reasonable request.
